# miR-18a as a potential regulator of estrogen-related genes in human breast carcinomas

**DOI:** 10.1186/1753-6561-7-S2-P59

**Published:** 2013-04-04

**Authors:** Renata C Bueno, Fabíola E Rosa, Francisco A Moraes-Neto, José Roberto F Caldeira, Sandra A Drigo, Silvia R Rogatto

**Affiliations:** 1 Department of Genetics, São Paulo State University, Botucatu, SP, Brazil; 2 Department of Pathology, São Paulo State University, Botucatu, SP, Brazil; 3 Department of Pathology, Amaral Carvalho Hospital, Jaú, SP, Brazil; 4 Department of Senology, Amaral Carvalho Hospital, Jaú, SP, Brazil; 5 NeoGene Laboratory, Department of Urology, São Paulo State University, Botucatu, SP, Brazil and A. C. Camargo Cancer Treatment and Research Center, São Paulo, SP, Brazil

## Background

Estrogen receptor (ER) status has been used as a biomarker in breast carcinomas (BC), allowing the identification of tumors that may respond to ER antagonists or aromatase inhibitors. Two previous studies by our group evaluating gene and miRNA expression profiles in BC samples from Brazilian patients revealed 15 genes and 3 microRNAs as significantly associated with BC, according to ER status. The aim of this study was to evaluate the correlation between these 3 microRNAs and ER-related genes in BC.

## Materials and methods

miRDIP algorithm was applied to select candidates among 15 ER-related genes for transcriptional regulation by hsa-miR-26a, hsa-miR-26b and miR-18a. Correlation analyses were performed between transcript levels of*ERRB4*, *TMEM205*, *SUSD3*, *DNAJC12*, *RERG*, *SCN7A*, *TBC1D9*, *TCEA3*, *THSD4*, *TIGD6* and miRNAs (miR-26a, miR-26b, and miR-18a) in BC compared with normal breast samples. miRNA expression was assessed in 63 BC and 5 normal breast samples by quantitative RT-PCR using as internal reference RNU48, RNU44 and U47.

## Results

Down-expression of miR-18a was significantly associated with ER-positive tumors (*P*=0.005), while down-expression of miR-26a and miR-26b were significantly associated with ER-negative tumors (*P*=0.006 and *P*=0.003, respectively). Additionally, a significant negative correlation was detected between overexpression of *ERBB4* and *TMEM205* and underexpression of miR-18a(*P*<0.001; r=-0.703 and *P*<0.001; r=-0.746, respectively).

## Conclusions

Our preliminary results suggest thatin ER-positive tumors, over-expression of *ERBB4* (receptor tyrosine kinase) and *TMEM205* (encoding a hypothetical protein associated with cisplatin resistance) genes could be explained by miR-18a down-expression. Functional studies are being conducted to confirm these findings. This study may contribute for the identification of potential therapeutic agents in BC.

## Financial support

CAPES and CNPq.

